# Neural inputs from spinal motor neurons to lateralis vastus muscle: Comparison between sprinters and nonathletes

**DOI:** 10.3389/fphys.2022.994857

**Published:** 2022-10-07

**Authors:** Fang Qiu, Xiaodong Liu, Yilin Xu, Lijun Shi, Xinjun Sheng, Chen Chen

**Affiliations:** ^1^ Department of Exercise Physiology, Beijing Sport University, Beijing, China; ^2^ School of Kinesiology, Shanghai University of Sport, Shanghai, China; ^3^ Sports Biomechanics Laboratory, Jiangsu Research Institute of Sports Science, Nanjing, China; ^4^ State Key Laboratory of Mechanical System and Vibration, Shanghai Jiao Tong University, Shanghai, China

**Keywords:** neural adaptation, motor unit, training, voluntary movements, electromyography decomposition

## Abstract

The adaptation of neural contractile properties has been observed in previous work. However, the neural changes on the motor unit (MU) level remain largely unknown. Voluntary movements are controlled through the precise activation of MU populations. In this work, we estimate the neural inputs from the spinal motor neurons to the muscles during isometric contractions and characterize the neural adaptation during training by comparing the MU properties decomposed from sprinters and nonathletes. Twenty subjects were recruited and divided into two groups. The high-density surface electromyography (EMG) signals were recorded from the lateralis vastus muscle during the isometric contraction of knee extension and were then decomposed into MU spike trains. Each MU’s action potentials and discharge properties were extracted for comparison across subject groups and tasks. A total of 1097 MUs were identified from all subjects. Results showed that the discharge rates and amplitudes of MUAPs from athletes were significantly higher than those from nonathletes. These results demonstrate the neural adaptations in physical training at the MU population level and indicate the great potential of EMG decomposition in physiological investigations.

## 1 Introduction

Voluntary movements are controlled through the precise activation of motor unit (MU) populations. As the smallest functional unit in the human neuromuscular system, the MU converts the descending neural inputs into forces to generate movements (Heckman and Enoka, 2012). The MU comprises two components, namely, the motor neuron and its innervated muscle fibers (Weinberger and Dostrovsky, 2010). These two components normally function as a single entity that provides the primary output for the central neural system. The ensemble of discharges of motor neurons innervating a muscle represents the neural drive transferred from the spinal cord to the muscle. It provides direct information on the functional tasks associated with muscle activation (Farina et al., 2013; Farina et al., 2014b; Dai et al., 2017). Moreover, information on MU behavior has contributed to a better understanding of the pathophysiological mechanisms of tremor (Holobar et al., 2012), stroke (Li et al., 2015), as well as the neural determinants of training and aging (Watanabe et al., 2016; Piasecki et al., 2019).

Neural and muscular adaptations are two of the most critical factors that contribute to the increase in muscle force caused by strength training. Narici et al. (1989) demonstrated that during strength training of the human quadriceps muscle, the increase in the anatomical cross-sectional area accounts for only 40% of the increase in force while the remaining 60% is attributable to an increased neural drive and possibly to architectural changes occurring within the muscle. The muscular and neural adaptations were also observed in dorsiflexor muscles (Van Cutsem et al., 1998). There were no changes in the time-to-peak torque and the recruitment sequence of the motor units, but obviously a different type of behavior occurs, since during ballistic contractions they were activated earlier in the contraction stage and their maximal firing rate was greater after training. de Ruiter et al. (2012) investigated the effect of imagery training on strength development and demonstrated the increase in contractile impulse only following physical training.

Previous studies have used transcranial magnetic stimulation or intramuscular recordings of electromyography (EMG) to investigate the types of neural adaptations elicited by strength training (Weier et al., 2012; Nuzzo et al., 2017). However, the neural changes on the MU level remain largely unknown. The human neural system modulates the recruitment/derecruitment and the discharge rate of MUs to realize precise force control. Previous studies have indicated that several weeks of strength training is sufficient in eliciting significant adaptations in the MU discharge rate and recruitment threshold force (Balshaw et al., 2016; Vecchio et al., 2018). These findings suggest that the adaptations in MU function may be attributable to changes in the synaptic input to the motor neuron pool or adaptations in intrinsic motor neuron properties (Vecchio et al., 2018). In addition, the muscle unit contractile properties may also change after long-term training. The number of motor neurons that innervate a muscle is comparatively stable, whereas the number of muscle fibers innervated by those motor neurons can increase (Heckman and Enoka, 2012).

Several studies have investigated the neuromuscular characters of sprinters. In Cristea et al. (2008) work, the 10% increase in squat jump in sprinters was accompanied by a 9% increase in the integrated EMG of the leg extensors. Kamandulis et al. (2012) found that sprint performance is poorly predicted by contractile kinetics of electrically induced contractions from the quadriceps muscle. Generally, the amplitude features, from whether intramuscular EMG or surface EMG, are used among in studies. However, the EMG features could only partially reflect the neural inputs from the spinal motor neurons. Up to now, there are few studies investigating the neural adaptations of sprinters at the MU level. The action potentials of MUs transfer to the surface of the skin and can be recorded as the surface EMG signals (Merletti and Farina, 2016). Therefore, they can be identified by processing and decomposing EMG signals, thereby allowing the spinal cord’s output to be accessed non-invasively (Del Vecchio et al., 2020).

MU activities can be decoded using electrodes placed inside the muscle or mounted on the skin surface. Needle/wire electrodes have been used for this purpose since the 1920s (Adrian and Bronk, 1929). Automatic algorithms for processing intramuscular signals were proposed in the 1970s (Farina et al., 2008; Merletti and Farina, 2009) and progressively improved over time (Negro et al., 2016; Roussel et al., 2017; Yu et al., 2020). However, intramuscular EMG (iEMG) can only identify relatively small samples of MUs, with fibers located close to the recording electrodes, because of the high selectivity. Alternatively, MU activities can be identified non-invasively by identifying the action potentials from interference surface EMG signals through blind source separation (Holobar and Zazula, 2007b; Holobar and Farina, 2014; Chen et al., 2018) or template matching with machine learning (Nawab et al., 2010; De Luca et al., 2015). The surface EMG recording modality allows the measurement of MU properties that are difficult to access with invasive recordings (e.g., muscle fiber conduction velocity or location of endplates). In addition, more MUs could be identified from surface EMG compared with iEMG signals (Merletti et al., 2008; Holobar et al., 2010; Holobar and Farina, 2014).

In the present study, we applied the EMG decomposition technique to analyzing MU activities of lateralis vastus muscle. The neural inputs sent into muscles during isometric contractions were estimated and characterized at the MU population level. Subsequently, the neural adaptations during training are interpreted by comparing the MU properties between sprinters and nonathletes.

## 2 Methods

### 2.1 Participants

A total of 12 sprinter athletes (Athlete group, all males, about 7 years sport-specific training) and 8 untrained subjects (Control group, all males, normal sports activity in school without further sports training) participated in the experiments. The athletes have all implemented specialized training (running rhythm, maximum speed, swing arm, etc.) and strength training (squat bench press, flip, jump, etc.). The detailed information of participants is shown in Table 1. All participants had no neurological disorders and had signed informed consent prior to the participation. The experimental protocol and the informed consents followed the Declaration of Helsinki.

**TABLE 1:** Anthropometric data of participants.

	Athletes (*n* = 12)	Non-athletes (*n* = 8)
Age (years)	20 ± 3	20 ± 2
Height (cm)	182 ± 5	178 ± 4
Body mass (kg)	70 ± 7	72 ± 8
BMI (kg/m^2^)	20.21 ± 1.42	22.12 ± 2.03

Values are mean ± standard deviation (SD). BMI: Body mass index. There were no significant differences between groups in the anthropometric data.

### 2.2 Experiments

#### 2.2.1 Experimental protocol

**FIGURE 1:**
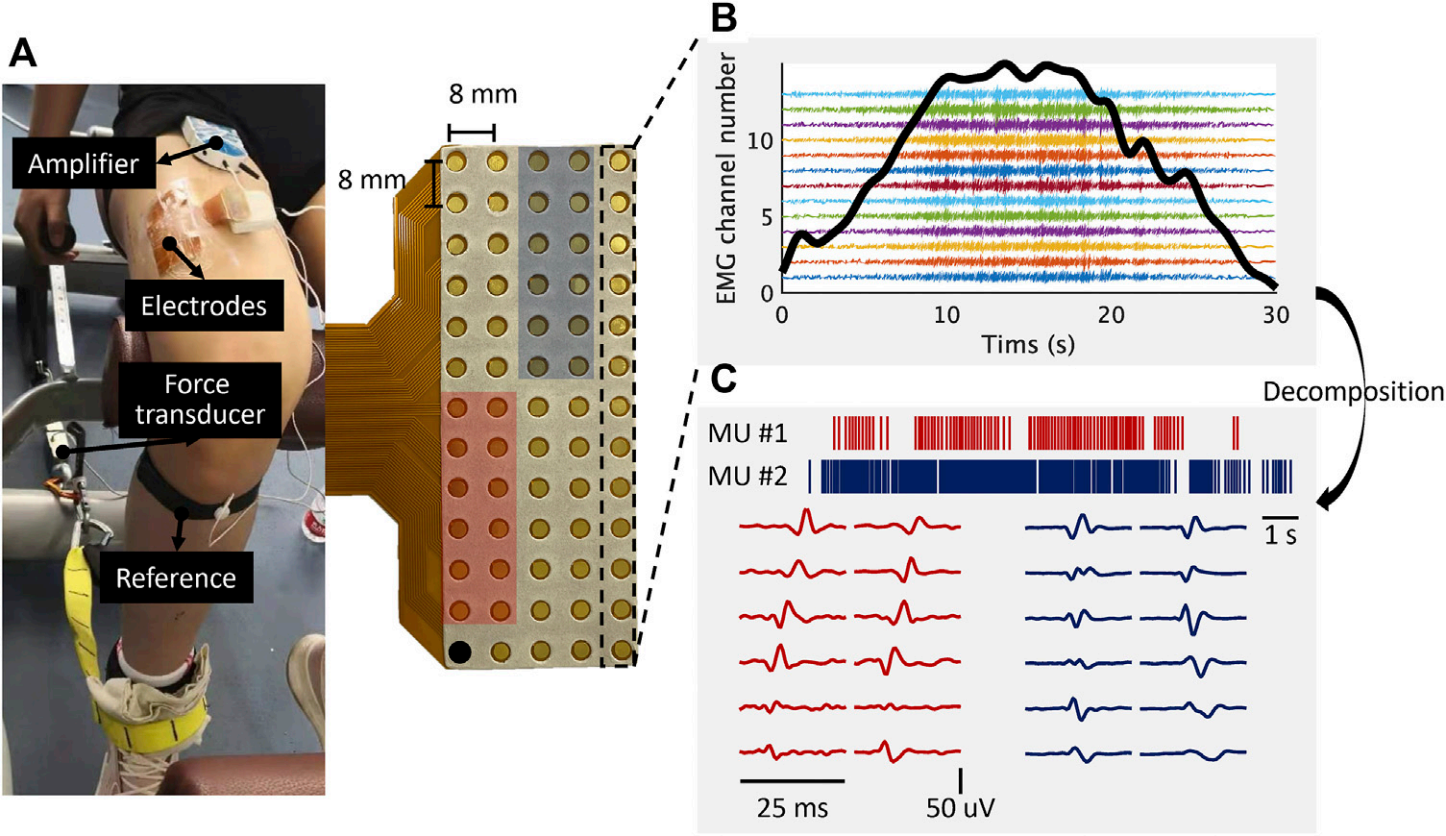
Experimental setup overview and EMG decomposition. **(A)**, a high-density electrode grid placed over the lateralis vastus muscles. **(B)**, the EMG signals from a column of the grid (13 channels). The bold black curve illustrates the force signal. The location of the EMG channels is indicated by the black rectangle. **(C)**, two representative MU spike trains and their multi-channel action potentials. The location of the action potentials is indicated by the red and blue blocks, respectively.

The subjects performed the static contraction of knee extension in the experiments. Before the experiments, the maximum voluntary contraction (MVC) of each subject was measured. The subjects were instructed to exert the maximum isometric contraction force of the knee extension three times, with a 1-min rest between each contraction. The average of the three extension forces was considered as the MVC force. The experiments involved two sessions. The subjects were instructed to perform knee extension by following a trapezoid force curve in each session. The trapezoid curve consisted of three phases. In session 1, the trapezoid curve lasted for 30 s, which included an ascending ramp of 10 s, a plateau phase of 10 s, and a descending ramp of 10 s (Figure 1B). The contraction level of the plateau phase was set as 30%, 50%, and 70% MVC. The subjects were instructed to perform one trial for each level. In session 2, the ascending/descending phase of the trapezoid curve was set as 2 s, whereas the plateau phase still lasted for 10 s. The other setup in session 2 was the same as that in session 1.

#### 2.2.2 Force signal recording

A custom-built force recording system was used to measure the knee kinetics in the experiments (Figure 1A). The force transducer (SM S-Type Load, Interface, United States) was mounted on the chair leg and arranged in series with a non-elastic strap (∼3 cm wide) to guarantee stiffness during the isometric contraction. Participants were comfortably seated with the hip flexed at ∼120° (180° = anatomical position) on a chair with the dominant knee extended at ∼90° (180° = anatomical position) and the ankle at ∼100° (90° = anatomical position) of plantar flexion. The strap was positioned over the distal portion of the foot dorsum, which was perpendicular to the tibia. The force transducer was amplified with a gain of 1015 and a sampling rate of 1000 Hz. The visual feedback of force signals was provided for subjects during the experiments.

#### 2.2.3 High-density electromyography recordings

The HDsEMG signals were recorded by an electrode grid with 64 channels (13 rows × 5 columns, ELSCH064NM2, OT Bioelettronica, Italy). The electrode diameter was 3 mm with an inter-electrode distance of 8 mm in both directions (Figure 1A). The electrode grids were mounted over the lateralis vastus muscle and connected with a multi-channel amplifier (Sessantaquattro, OT Bioelettronica, Italy). The HDsEMG signals were recorded in a monopolar mode with a gain of 1,000 and a sampling rate of 2,000 Hz. The EMG signals were bandpass filtered between 3 Hz and 900 Hz by hardware and A/D converted on 12 bits. The EMG data were recorded wirelessly with the OT BioLab (OT Bioelettronica, Italy) and preserved for offline analysis.

### 2.3 Data analysis

**FIGURE 2:**
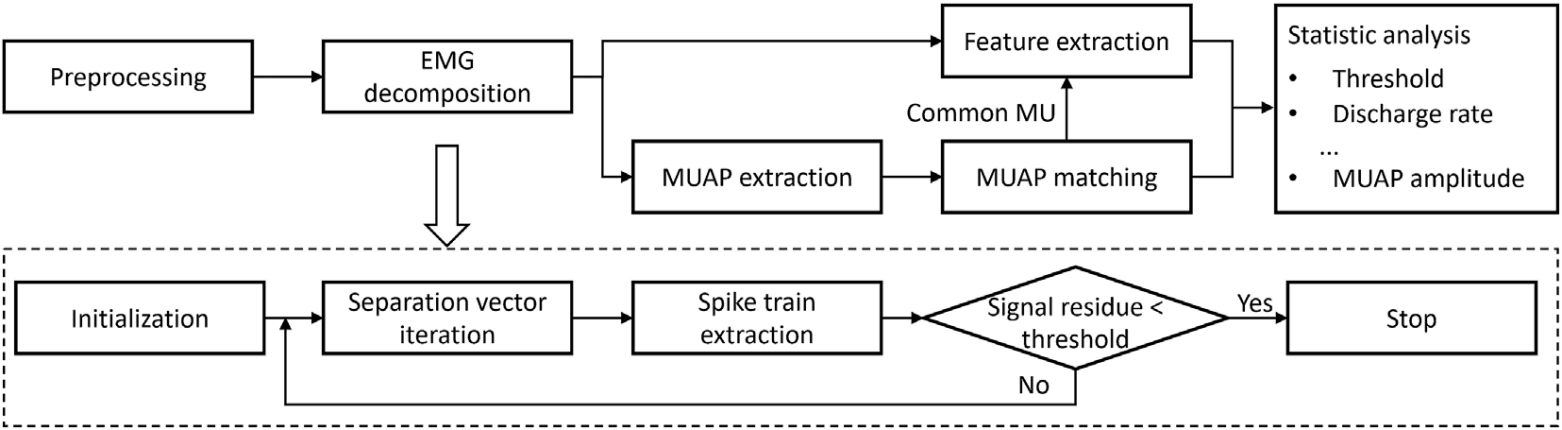
The flowchart of data analysis and EMG decomposition.

The flowchart of data analysis and decomposition procedure is illustrated in Figure 2. First, the EMG signals were preprocessed and decomposed into MU spike trains (MUSTs). Then the MU action potentials (MUAPs) were extracted to identify common MUs. Several neural contractile properties were extracted from the MUSTs and MUAPs.

#### 2.3.1 Preprocessing

The force signals were up-sampled at 2000 Hz for the subsequent analysis. Some EMG channels (usually less than 5) were discarded by visual inspection due to excessive noise caused by poor contact between the skin and electrodes or electromagnetic interference. A fourth-order Butterworth bandpass filter (20–500Hz) was applied to all the remaining channels. Another comb filter with a cutoff frequency of 50 Hz was used to remove the power line interference.

#### 2.3.2 Electromyography decomposition

The surface EMG signals were decomposed into MUSTs using the convolution kernel compensation (CKC) algorithm (Holobar and Zazula, 2007a). The decomposition algorithm is described in detail in (Holobar and Zazula, 2007a), and a brief explanation of the decomposition basis is provided here.

The generation model of multi-channel EMG signals can be described as a convolutive mixture of a series of impulses (MUST) and their responses (Holobar and Zazula, 2007a; Negro et al., 2016). The impulse responses in this mixture model are the action potentials of the MUs, which have a finite duration (Holobar and Zazula, 2007a; Holobar and Zazula, 2007b).

$$x_{i}\left(n\right)=\overset{N}{\underset{j=1}{\sum }}\overset{L-1}{\underset{l=0}{\sum }}h_{ij}\left(l\right)s_{j}\left(n-l\right)+\omega_{i}\left(n\right),\;i=1,2,\ldots ,M$$ (1)

where $x_{i}\left(n\right)$ is the i th EMG channel, n is the discrete time as sample point, $h_{ij}\left(l\right)$ is the action potential of the j th MU recorded at the channel i, $s_{j}\left(n\right)$ is the spike train of the j th MU (the j th MUST), $\omega_{i}\left(n\right)$ is the additive noise at channel i , L is the sample length of the action potentials, N and M is the number of active MUs and EMG channels, respectively. The Eq. 1 can be converted to matrix form:

$$x\left(n\right)=H\bar{s}+\omega \left(n\right)$$ (2)

where $\omega \left(n\right)={\left[\omega_{1}\left(n\right),...,\omega_{M}\left(n\right)\right]}^{T}$ is the addictive noise, $\bar{s}\left(n\right)={\left[s_{1}\left(n\right),s_{1}\left(n-1\right),...,s_{1}\left(n-L+1\right),...,s_{N}\left(n\right),s_{N}\left(n-1\right),...,s_{N}\left(n-L+1\right)\right]}^{T}$ is the extended version of vector from N sources $s\left(n\right)={\left[s_{1}\left(n\right),...,s_{N}\left(n\right)\right]}^{T}$ , and the mixing matrix H is:

$$H=\left[\begin{array}{cc} \begin{array}{ccc} h_{11}\left(0\right) & \cdots & h_{11}\left(L-1\right)\\ h_{21}\left(0\right) & \cdots & h_{21}\left(L-1\right)\\ \vdots & \cdots & \vdots \end{array} & \begin{array}{ccc} h_{12}\left(0\right) & \cdots & h_{1N}\left(L-1\right)\\ h_{22}\left(0\right) & \cdots & h_{2N}\left(L-1\right)\\ \vdots & \cdots & \vdots \end{array}\\ \begin{array}{ccc} h_{M1}\left(0\right) & \cdots & h_{M1}\left(L-1\right) \end{array} & \begin{array}{ccc} h_{M2}\left(0\right) & \cdots & h_{MN}\left(L-1\right) \end{array} \end{array}\right]$$ (3)

The CKC method compensates the unknown mixing matrix *H* in Eq. 2 and estimates the spike train of the j th MU (Holobar and Zazula, 2007a):

$$\hat{s_{j}}\left(n\right)=c_{s_{j}x}^{T}C_{xx}^{-1}x\left(n\right)$$ (4)

where $C_{xx}=E\left(x\left(n\right)x^{T}\left(n\right)\right)$ is the correlation matrix of EMG signals, $c_{s_{j}x}=E\left(x\left(n\right)s_{j}^{T}\left(n\right)\right)$ is the cross-correlation vector, and E(·) denotes mathematical expectation.

Suppose $w_{j}=C_{xx}^{-1}{\hat{c}}_{s_{j}x}$ , where ${\hat{c}}_{s_{j}x}$ is the estimation of $c_{s_{j}x}$ (Holobar and Zazula, 2007a), the estimation of spike train (Eq. 4) can be written as:

$${\hat{s}}_{j}\left(n\right)=w^{T}x\left(n\right)$$ (5)

where $w_{j}$ is the estimation of the j th separation vector. The estimation of the separation vector can be realized through iteration steps using the natural gradient descent algorithm (Holobar and Zazula, 2007a). Then the estimation of spike trains of the j th MU can be calculated depending on Eq. 5. The MU discharges were extracted using the K-means clustering method from the estimated spike train. After the j th MUST is extracted, the repeating procedure of MUST extraction is implemented on the residue signals until the root mean square value of the residue signal is lower than the threshold set manually.

A signal-based performance measure called pulse-to-noise ratio (PNR) (Holobar et al., 2014) was used to evaluate the decomposition accuracy of MUSTs. There is a strong correlation between this indicator and the decomposition’s sensitivity and false alarm rate (Holobar et al., 2014; Negro et al., 2016). Thus, PNR is a reliable and robust assessment of accuracy in identifying MUSTs by using the CKC-based algorithm (Holobar et al., 2014). In this study, MUSTs with PNR < 20 dB were discarded in the subsequent analysis because of the low confidence of decomposition accuracy. The discharges separated by more than 1 s (in both forward and backward direction) were also excluded and most likely mis-identified.

#### 2.3.3 MU action potential extraction and matching

The EMG signals were bipolar-filtered along the muscle fiber direction before MUAP extraction. Subsequently, the multi-channel MUAP waveforms of each MU were obtained by spike-triggered averaging (Farina et al., 2010). For each channel, signals from a 25 ms interval, which were triggered by the discharge timings of each MU, were collected and averaged.

The MUAP waveforms were used to match the same MU identified across different trials (Kapelner et al., 2017; Martinez-Valdes et al., 2017). At least 10% of channels with the maximum peak amplitude of waveforms were selected. The normalized cross-correlation coefficients were calculated between MUAP waveforms from paired channels. Two MUs were matched if the average correlation coefficient of all paired channels was higher than 0.7 and the average difference in peak amplitude for all paired channels was less than 30%. Only the MUs tracked across two or more trials were preserved for the following analysis. These common MUs were regarded comparatively reliable since it is less likely to misidentify an MU more than once.

#### 2.3.4 Feature extraction

The following MU properties were extracted from MUSTs and MUAPs for the comparison of subjects and tasks.

• Recruitment threshold refers to the contraction level when a MU is recruited. It is also defined as the contraction level that corresponds to the first spike.
• Derecruitment threshold refers to the contraction level when a MU is derecruited. It is also defined as the contraction level that corresponds to the last spike.
• Recruitment discharge rate refers to the average discharge rate at the recruitment phase. It is also defined as the reciprocal of the average value of the first five inter-spike intervals (ISI).
• Stable discharge rate refers to the average discharge rate at the plateau phase. It is also defined as the reciprocal of the average value of the five middle ISIs (in the plateau phase).
• Decruitment discharge rate refers to the average discharge rate at the derecruitment phase. It is also defined as the reciprocal of the average value of the last five ISIs.
• Peak-to-peak value (PPV) refers to the maximum PPV of MUAP waveforms among all channels.

The cumulative spike train (CST) was also calculated by combining all the MUSTs. The correlation analysis was implemented between the discharge rate of CST and the force of the knee extension to estimate the neural drive to the muscles. To avoid the influence caused by the quantity of identified MUs, the number of MUs used to calculate the CST was fixed to 5. In each trial, 5 MUs were randomly selected for 100 times. The correlation in each trial was measured by averaging the Pearson correlation coefficient (R) across 100 times.

All the decompositions and data analysis were implemented in MATLAB 2021b (Matlab Inc. United States).

### 2.4 Statistics

The factor (independent variable) corresponds to subject groups (sprinter athletes and untrained subjects) or protocols (10s-ramp tasks and 2s-ramp tasks). Dependent variables refer to the MU properties. Before performing the analysis of variance (ANOVA), the homogeneity of variance for all the data was tested first. If satisfied, then the Bonferroni method was conducted. If not, then the Dunnett’s C method was used instead. The significance level (*p*) was set to 0.05. Symbols ∗, ∗∗, ∗∗∗ indicate significant differences with a level of (0.01 < *p* < 0.05), (0.001 ≤ *p* ≤ 0.01), (*p* < 0.001), respectively.

## 3 Results

**FIGURE 3:**
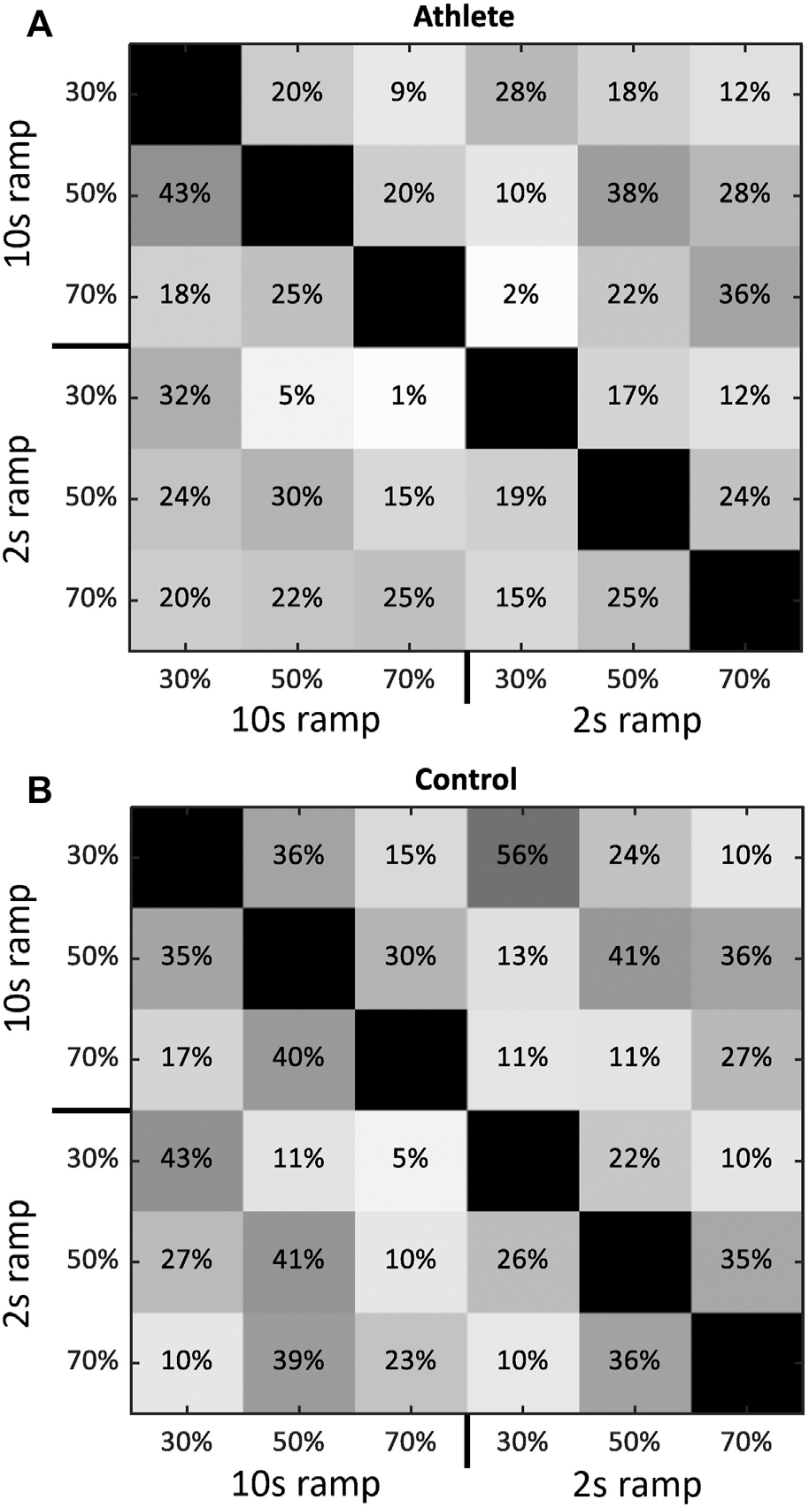
The matching results of MU action potentials across tasks. **(A,B)** shows the results from Athlete and Control group, respectively. Each block gives the matched rate of MUs between the corresponding task in the vertical and horizontal axis averaged across subjects. The matching rates in the diagonal blocks are 100%.

The total number of identified MUs was 1097 (636 for the Athlete group, 461 for the Control group). On average, there were 54 ± 35 MUs decoded from each subject and 9 ± 7 from each trial. The number of common MUs that were matched at least once was 615 (317 for the Athlete group, 298 for the Control group). The average PNR in each trial was higher than 30 dB, thereby suggesting the decomposition sensitivity of approximately *>*85% and a false alarm rate of *<*2% (Holobar et al., 2014). Table 2 shows the summary of decomposition results in each trial. Figure 3 illustrates the average matching results across subjects.

**TABLE 2:** Summary of decomposition results.

Group	Session (s)	Level (%MVC)	Number of MUs		PNR (dB)	Thresholds (%MVC)		PPV (mV)	Discharge rate (Hz)		
			All	Common		Recruitment	Derecruitment		Recruitment	Stable	Derecruitment
Athlete	10	30	12 ± 4	6 ± 4	32.29 ± 2.73	15.49 ± 9.07	12.36 ± 11.19	0.19 ± 0.08	8.52 ± 2.73	7.98 ± 6.91	7.22 ± 2.77
		50	7 ± 5	4 ± 4	31.59 ± 3.08	32.47 ± 11.77	25.56 ± 18.72	0.28 ± 0.08	10.13 ± 3.96	10.75 ± 8.43	8.58 ± 4.29
		70	6 ± 4	3 ± 2	31.22 ± 3.12	42.36 ± 19.15	35.05 ± 25.67	0.38 ± 0.11	9.68 ± 4.39	10.43 ± 8.77	8.99 ± 3.33
	2	30	10 ± 8	4 ± 4	32.50 ± 2.14	20.77 ± 8.83	13.58 ± 11.46	0.20 ± 0.09	11.66 ± 5.65	7.07 ± 5.76	9.79 ± 2.96
		50	10 ± 6	5 ± 3	31.43 ± 2.37	32.66 ± 18.30	26.39 ± 17.96	0.29 ± 0.09	12.17 ± 4.56	9.85 ± 7.74	11.87 ± 4.46
		70	9 ± 7	4 ± 3	30.72 ± 3.02	49.74 ± 18.25	34.71 ± 26.23	0.38 ± 0.10	14.64 ± 5.39	9.44 ± 7.87	13.21 ± 4.92
Control	10	30	10 ± 8	7 ± 5	30.42 ± 4.00	20.50 ± 8.84	17.40 ± 8.81	0.11 ± 0.05	7.26 ± 3.33	12.13 ± 7.21	6.85 ± 3.97
		50	9 ± 7	6 ± 4	30.91 ± 2.95	33.81 ± 10.74	31.51 ± 12.51	0.17 ± 0.06	8.44 ± 4.29	11.42 ± 6.98	6.60 ± 3.42
		70	7 ± 6	5 ± 5	31.38 ± 2.31	51.19 ± 13.79	51.16 ± 14.16	0.23 ± 0.07	8.69 ± 3.39	10.76 ± 4.54	7.54 ± 4.08
	2	30	11 ± 8	7 ± 6	30.84 ± 2.88	21.21 ± 8.75	17.84 ± 8.68	0.12 ± 0.05	9.02 ± 3.93	10.95 ± 6.78	8.18 ± 3.52
		50	10 ± 9	7 ± 6	31.37 ± 3.52	38.24 ± 9.99	32.72 ± 11.79	0.17 ± 0.08	10.79 ± 4.82	11.22 ± 6.49	9.53 ± 3.91
		70	11 ± 8	6 ± 5	30.69 ± 3.51	57.34 ± 12.00	47.89 ± 16.09	0.22 ± 0.08	12.62 ± 6.37	12.95 ± 6.47	11.28 ± 6.86

**FIGURE 4:**
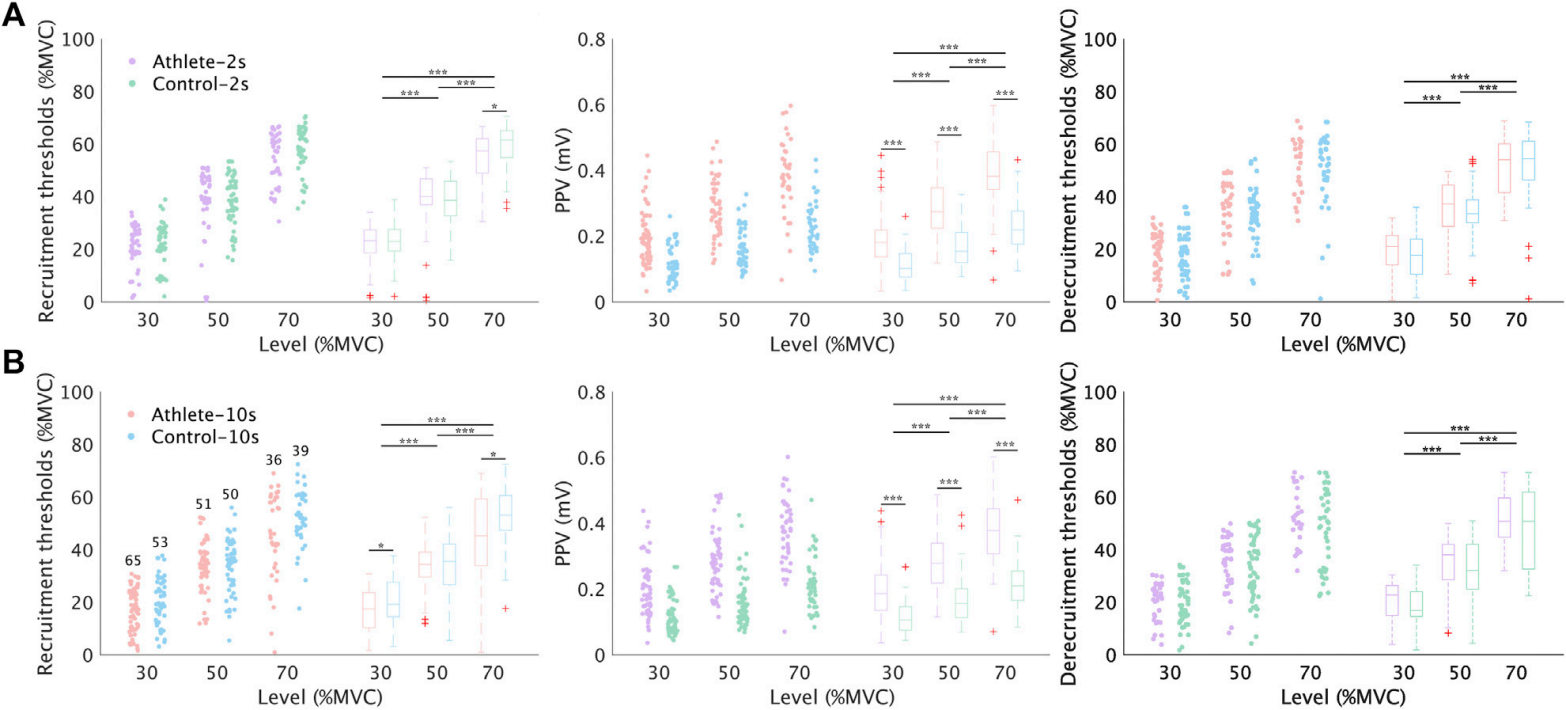
Comparison of recruitment threshold, derecruitment threshold, and MUAP amplitudes between Athlete and Control group. **(A,B)** illustrate the comparison results from session 1 and session 2, respectively. Only the common MUs were analyzed in this figure. The number of common MUs is indicated in the left subgraph. The scatters show all the values of each metric, while the box plots show the corresponding statistic distribution. The significance level of the difference between groups or contraction levels is provided above the box plots.

**FIGURE 5:**
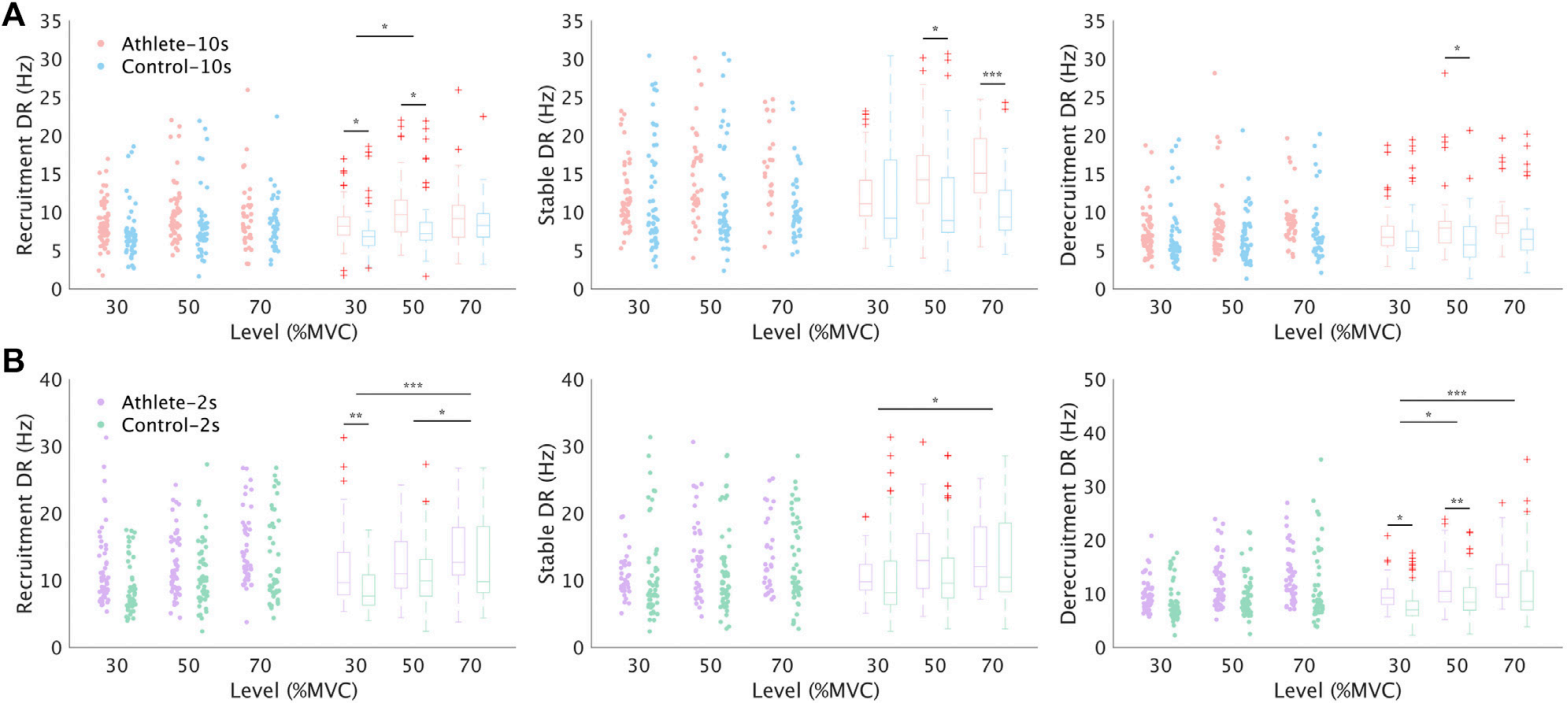
Comparison of MU discharge rate between Athlete and Control group. **(A,B)** illustrate the comparison results from session 1 and session 2, respectively. Only the common MUs, which were the same as in Figure 4, were analyzed in this figure. DR: discharge rate. The representation of symbols are the same as those in Figure 4.

The comparison results for the MU properties between subject groups were demonstrated in Figures 4, 5. Figure 4 illustrates the properties related to recruitment and MU size. A few MUs (usually *<*1 MU for each trial) were discarded due to the abnormal discharge properties. No significant difference was observed in the recruitment or derecruitment thresholds between the Athlete and Control groups during nearly all conditions. Meanwhile, the PPV of MUAP from the Athlete group was dramatically higher than that in the Control group. In addition, the contraction level affected these three properties in both groups. Generally, the (de) recruitment threshold and the PPV increased with the contraction level.

**FIGURE 6:**
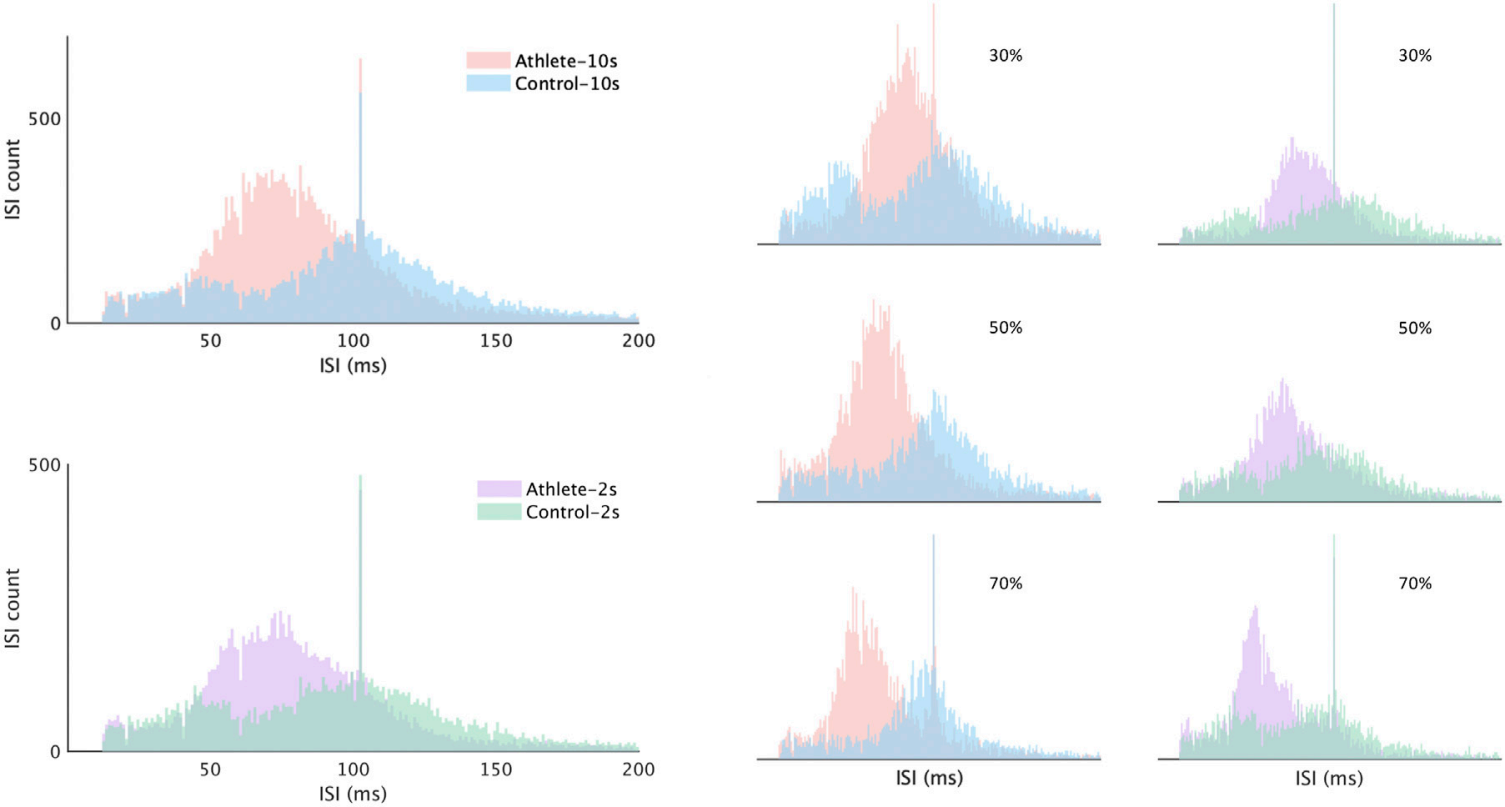
Distribution of MU interspike intervals. Histograms of MU interspike intervals are shown for the Athlete and Control group from each level and session. The interspike intervals were calculated from the entire contractions including ascending, plateau, and descending phases.

Figure 5 shows the comparison results of discharge rate properties. In nearly all conditions, except for one (Figure 5B, middle graph, level 30%), the average discharge rate of the Athlete group was higher than that in the Control group. Approximately half of the conditions showed significance. The difference in discharge rate between subject groups is also clearly presented in Figure 6. The distributions of interspike intervals exhibited significant difference when grouping all subjects and levels in each group. The distribution during each session and level is also shown in Figure 6.

**FIGURE 7:**
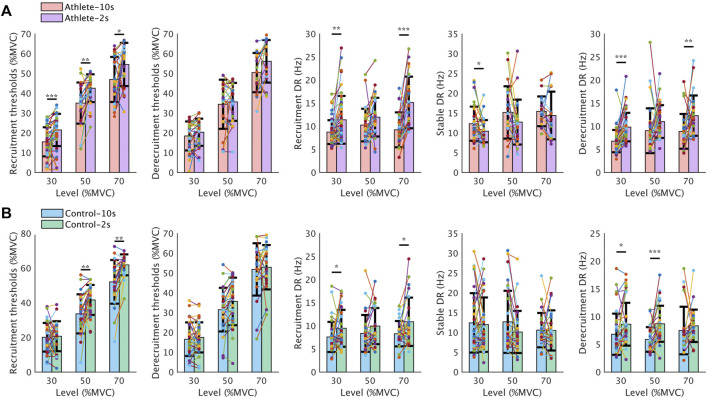
Comparison of recruitment threshold, derecruitment threshold, and discharge rate between 10s-ramp and 2s-ramp tasks. The scatter plots indicate all the values of each metric from the common MUs (filled circles) tracked between different tasks (solid lines). Each common MU is indicated by a different color, and the solid line between two points indicates the variation trend of the same MU. The bars show the average values in each task. **(A,B)** illustrate the comparison results from the Athlete and Control groups, respectively. Data are reported as the mean ± SD. DR: discharge rate.

Figure 7 shows the comparison results for the five MU properties across sessions. The PPV is not shown here because of the natural consistency of MUAP amplitudes for MU matching. The average recruitment threshold of MUs exhibited an increase of about 10% MVC when the tasks changed from 10s-ramp to 2s-ramp (*p <* 0.05), while there was no significant change in the derecruitment threshold. As to the discharge rate, the MUs in 2s-ramp sessions usually discharged with a higher rate in all three phases (recruitment, stable, derecruitment).

**FIGURE 8:**
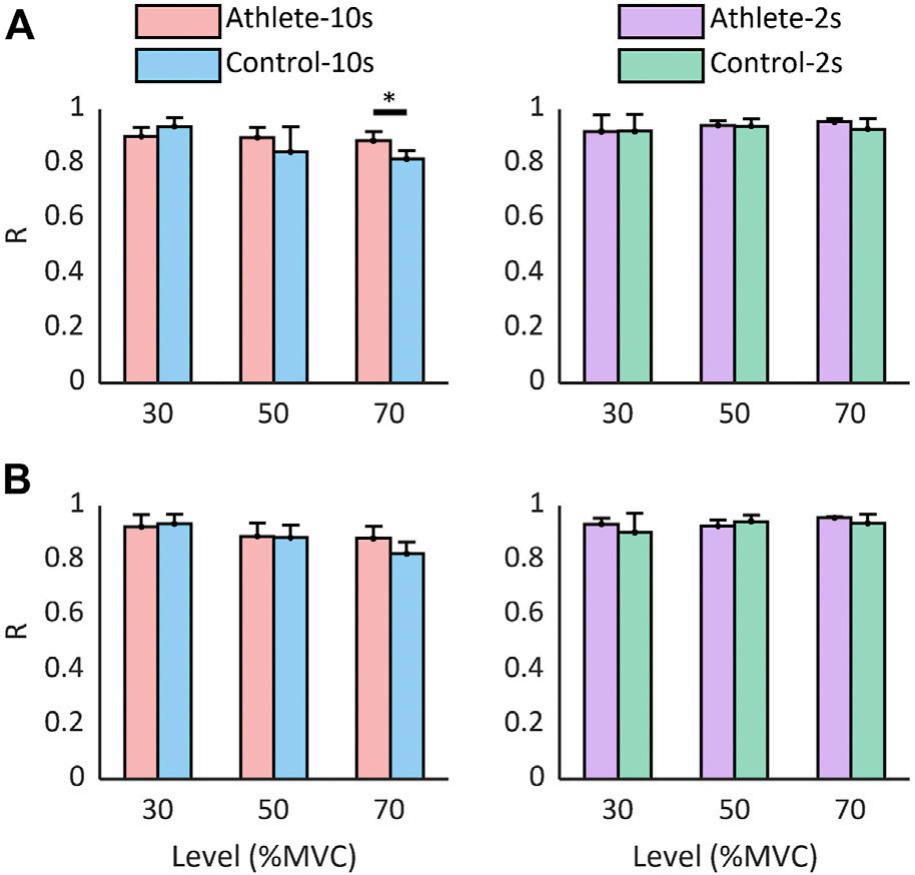
The correlation between force and discharge rate of cumulative spike trains. The correlation analysis was performed using CSTs calculated based on all MUs **(A)** and common MUs **(B)**. Data are reported as the mean ± SD.

Figure 8 shows the correlation between the discharge rate of cumulative spike trains and force. The average correlation coefficient in each trial was higher than 0.9, and there was no significant difference in most trials.

## 4 Discussion

In this study, we characterized the behavior of a relatively large population of MUs during voluntary isometric contractions. The increase in discharge rate and action potential amplitudes was observed at the MU population level. The results demonstrate the impact of training on the rate coding and contractile properties of MUs, providing compelling evidence for the adaptation of neural inputs.

### 4.1 Decoding neural drive based on electromyography decomposition

EMG decomposition technique provides a non-invasive way to decode the neural outputs from the spinal cord. On the one hand, the MUSTs, which refer to the series of discharge timings of the motor neuron, directly reflect the neurophysiological process underlying the muscle contraction (Farina et al., 2014a). On the other hand, the MUAPs, which correspond to the sum of muscle fiber potentials, are highly correlated with the contractile properties of muscles (Heckman and Enoka, 2012). In the present study, we decomposed the EMG signals into MUSTs using a blind source separation algorithm and extracted the MUAPs from interference EMG signals, thus allowing the direct analysis of neural adaptation for single MUs.

Although the PNR threshold to select MUSTs was set as 20 dB, the average PNR in each trial was higher than 30 dB, which indicates the high accuracy of the EMG decomposition. The value of this index is comparable with previous investigations (Holobar et al., 2014). After decomposition, the multi-channel action potentials of each MU were obtained through post-processing and used for MU tracking across trials. The matching method based on the correlation of waveforms has been validated and applied in the long-term tracking of MUs (Martinez-Valdes et al., 2017; Del Vecchio et al., 2019a). Over half of the MUs were successfully tracked across trials. These results indicate high decomposition accuracy because it is very unlikely that two independent decompositions make the same errors for different trials. On the contrary, the non-tracked MUs do not necessarily indicate errors. Notably, all the MU activities in a muscle can impossibly be identified because of the limitation of the current decomposition methods (Negro et al., 2016). The number of the identified MUs is affected by the decomposition parameters (e.g., the number of iteration and the loop, the preprocessing methods) and the complexity of EMG signals (the amount of activating MUs and the power of noise) (Holobar and Farina, 2014; Chen et al., 2020). Therefore, identifying different populations of MUs in two trials was normal.

### 4.2 Neural adaptation during training

The properties of motor neurons (e.g., recruitment threshold, discharge rate) and contractile apparatus (e.g., number of muscle fibers, time to peak force) can be influenced by training. Among these properties, the recruitment order is relatively resistant to changes and is not influenced by interventions, such as aging (Klass et al., 2008; Fling et al., 2009), or physical training (Van Cutsem et al., 1998). Significant difference in the (de)recruitment threshold was not observed between the Athlete and Control groups in the present study. By using the EMG decomposition technique, we observed the similar neural activities of MUs as in previous studies. In addition, the contraction level dramatically affected the distribution of (de)recruitment thresholds, which was due to the fact that only a fraction of MU populations were decoded from EMG signals.

The discharge rate in each contraction phase was significantly influenced by training. Previous studies suggest that the discharge rate appears to decline with age and contribute to age-associated decreases in muscle strength (Barry et al., 2007; Knight and Kamen, 2008) but will increase in response to strength training in young and old humans (Kamen and Knight, 2004). The discharge rates in the Athlete group were higher than those in the Control group in nearly all the conditions, even though no significance was shown in a few conditions. The difference in discharge rate can be observed more clearly in the distribution of interspike intervals (Figure 6). In all the conditions with different contraction speeds and levels, the center of the ISI distribution from the Athlete group was much lower than that from the Control group, thereby indicating the increase in discharge rates with training.

Apart from the neural adaptations, muscular adaptations, such as the hypertrophy of muscle fibers, also contribute to the strength increase in physical training and have a different time course throughout the training process (Cristea et al., 2008; Duchateau and Baudry, 2010). In this study, we evaluate the muscular adaptations indirectly by analyzing the MUAP amplitudes. The MU size, including the number of muscle fibers innervated by each motor neuron and their average cross-sectional area, was indirectly estimated by the PPV of the MUAP waveform. The increase in PPV indicated that the strength rise after training could result from the hypertrophy of existing muscle fibers as well as from an increased number of fibers (Narici et al., 1989). However, it should be noted that the PPV of MUs may be affected by a couple of factors rather than training, such as the thickness of the fat layer. The thick fat layer attenuates the waveform, resulting in a lower PPV.

During an isometric contraction, the ramp increase and decrease in force to a submaximal target, which involves the concurrent recruitment and rate modulation of MUs (Del Vecchio et al., 2019b). On the one hand, it was suggested that rapid contractions might involve the preferential recruitment of fast-contracting MUs, which may explain the difference in recruitment threshold change in the two sessions with different contraction speeds. Different populations of the MUs are recruited at first when contracting faster. Therefore, the recruitment of MUs in the 10 s-ramp session was postponed. On the other hand, rapid contractions are also characterized by an increase in discharge rate (Figure 7), thereby demonstrating the effect of rate modulation in rapid contraction (Heckman and Enoka, 2012).

### 4.3 Neural drive to muscle and the force

Although the current decomposition methods could only identify a small population of activating MUs, we can obtain the accurate estimation of neural drive to the muscle by pooling the MUs (Negro et al., 2009). The CST feature has been demonstrated to represent the common drive transferred from the spinal cord into the muscle (Farina et al., 2014b; Holobar and Glaser, 2019). Therefore, the CST is highly correlated with the contraction force of a single muscle. By pooling the MUs together and extracting the CST feature, we obtained a reliable estimate of the neural commands transferred to the muscle, which has a great potential in the muscle activation estimation and kinetics projection. The muscle synergies can also be evaluated using the decomposition technique (Tanzarella et al., 2021). Moreover, the muscle activation estimation based on EMG decomposition is robust to the contraction speed and training because no significant difference in the correlation between CST and muscle was observed in most conditions.

### 4.4 Future work

In this work, the adaptations in neural inputs were only characterized based on the identified motor units. Several techniques, such as the H reflex test, could also be used to evaluate the changes in spinal reflex dynamics and amplitudes. Further validation of the neural adaptations are needed in the future work. As the MU discharges and their action potentials could be identified, the EMG decomposition has a great potential to investigate the nervous control and the characteristics of this control, such as the vector encoding of the motor response and the effect of the possible sensory information’ anticipation generated by the movement itself. Apart from the neural adaptations, the muscular adaptations require to be directly analyzed in the future work, aiming to interpret better the physiological mechanism underlying strength training.

## 5 Conclusion

The neural adaptation of training was investigated by characterizing the properties of MU from sprinters and nonathletes. The training increased the discharge rates and action potential amplitudes during the same force level. The results from the present study demonstrate the impact of training on the rate coding and contractile adaptation at the MU population level. These results provide compelling evidence that the output from the spinal cord during prescribed actions is augmented after long-term training.

## Data Availability

The raw data supporting the conclusions of this article will be made available by the authors, without undue reservation.
